# Development and Evaluation of a Machine Learning Prediction Model for Small-for-Gestational-Age Births in Women Exposed to Radiation before Pregnancy

**DOI:** 10.3390/jpm12040550

**Published:** 2022-03-31

**Authors:** Xi Bai, Zhibo Zhou, Yunyun Luo, Hongbo Yang, Huijuan Zhu, Shi Chen, Hui Pan

**Affiliations:** Key Laboratory of Endocrinology of National Health Commission, Department of Endocrinology, State Key Laboratory of Complex Severe and Rare Diseases, Peking Union Medical College Hospital, Chinese Academy of Medical Science and Peking Union Medical College, Beijing 100730, China; baixi199532@163.com (X.B.); pumc_zhouzhibo@student.pumc.edu.cn (Z.Z.); pumc_luoyunyun@student.pumc.edu.cn (Y.L.); yanghb@pumch.cn (H.Y.); shengxin2004@163.com (H.Z.); cspumch@163.com (S.C.)

**Keywords:** small for gestational age, exposure to radiation, machine learning, prediction

## Abstract

Exposure to radiation has been associated with increased risk of delivering small-for-gestational-age (SGA) newborns. There are no tools to predict SGA newborns in pregnant women exposed to radiation before pregnancy. Here, we aimed to develop an array of machine learning (ML) models to predict SGA newborns in women exposed to radiation before pregnancy. Patients’ data was obtained from the National Free Preconception Health Examination Project from 2010 to 2012. The data were randomly divided into a training dataset (*n* = 364) and a testing dataset (*n* = 91). Eight various ML models were compared for solving the binary classification of SGA prediction, followed by a post hoc explainability based on the SHAP model to identify and interpret the most important features that contribute to the prediction outcome. A total of 455 newborns were included, with the occurrence of 60 SGA births (13.2%). Overall, the model obtained by extreme gradient boosting (XGBoost) achieved the highest area under the receiver-operating-characteristic curve (AUC) in the testing set (0.844, 95% confidence interval (CI): 0.713–0.974). All models showed satisfied AUCs, except for the logistic regression model (AUC: 0.561, 95% CI: 0.355–0.768). After feature selection by recursive feature elimination (RFE), 15 features were included in the final prediction model using the XGBoost algorithm, with an AUC of 0.821 (95% CI: 0.650–0.993). ML algorithms can generate robust models to predict SGA newborns in pregnant women exposed to radiation before pregnancy, which may thus be used as a prediction tool for SGA newborns in high-risk pregnant women.

## 1. Introduction

Small-for-gestational-age (SGA) neonate is defined as a birth weight below a distribution-based gestational age threshold, usually the 10th percentile [1]. SGA newborns are at increased risk of perinatal morbidity and mortality [2,3]. The main risk factor related to stillbirth is unrecognized SGA before birth [4]. However, if the condition is identified before delivery, the risk can be substantially reduced, even a four-fold reduction, because antenatal prediction of SGA allows for closer monitoring and timely delivery to reduce adverse fetal outcomes [2].

Environmental pollutants have been associated with adverse pregnancy outcomes and a reduction in birth weight [5,6,7]. Human and animal studies have shown that the proportion of SGA increases with exposure to radiation [8,9]. High-level radiation exposure produced SGA neonates in the offspring of pregnant atomic bomb survivors [10]. Additionally, it has been reported that the radiation exposure rate in mothers with low-birth-weight newborns was higher than those with normal weight newborns [11]. Even data from studies has demonstrated that each cGy radiation reduced the birth weight of newborns by 37.6 g [12]. The causes have been reported to be the effects of radiation on the function of the ovary and uterus, as well as the effect on the hypothalamus–pituitary–thyroid axis [13,14]. However, no study has established a predictive model for SGA newborns in women exposed to radiation before pregnancy.

Risk predictive models relying on conventional statistical methods affect their application and performance in large datasets with multiple variables due to the inherent limitations of not considering the potential interactions between risk factors [15,16]. However, these limitations can be solved by machine learning (ML) approaches which can model complex interactions and maximize prediction accuracy from complex data [17]. In terms of SGA risk prediction, ML algorithms have been introduced into a few studies to obtain predictive models for SGA in the general population [18,19,20]. Unfortunately, these models performed poorly, with the maximum area under the receiver operating characteristic (ROC) curve (AUC) value as high as only 0.7+. In addition, paternal risk factors and maternal PM2.5 exposure during pregnancy have been confirmed as risk factors for SGA newborns [21,22,23]. Although these independent risk factors are identified, they have not been included in previous predictive models.

In this report, we aimed to develop and validate models using different ML algorithms to predict SGA newborns in pregnant women exposed to radiation in a living or working environment before pregnancy, based on data from a nationwide, prospective cohort study in China. In addition, paternal risk factors and pregnancy PM2.5 exposure were innovatively included in the models as predictive features.

## 2. Materials and Methods

### 2.1. Data Source

Data were obtained from the National Free Preconception Health Examination Project (NFPHEP), a 3-year project from 1 January 2010 to 31 December 2012, which was carried out in 220 counties from 31 provinces or municipalities and initiated by the National Health Commission of the People’s Republic of China [24,25,26]. In short, the NFPHEP aimed to investigate risk factors for adverse pregnancy outcomes and improve the health of pregnant women and newborns. All data were uploaded to the nationwide electronic data acquisition system, and quality control was carried out by the National Quality Inspection Center for Family Planning Techniques. This study was approved by the Institutional Review Committee of the National Research Institute for Family Planning in Beijing, China, and informed consent was obtained from all participants.

### 2.2. Study Participants and Features

All singleton live newborns with complete birth records and gestational age of more than 24 weeks were included in the study, and then we selected newborns whose mothers were exposed to radiation in their living or working environment before pregnancy, involving 985 cases. After removing records with missing and extreme data of baseline characteristics, 455 births were included in the final analysis.

A pre-pregnancy examination was conducted, and follow-up was performed during pregnancy and postpartum. Information of 153 features regarding parents’ social demographic characteristics, lifestyle, family history, pre-existing medical conditions, laboratory examinations and neonatal birth information were collected through face-to-face investigation and examination performed by trained and qualified staff. PM2.5 concentrations for all included counties were provided by the Chinese Center for Disease Control and Prevention, using a hindcast model specific to historical PM2.5 estimation provided by satellite-retrieved aerosol optical depth [27]. The definition of SGA was newborns with a birth weight below the 10th percentile for the gestational age and sex according to the Chinese Neonatal Network [28].

### 2.3. Study Design

**Figure 1:**
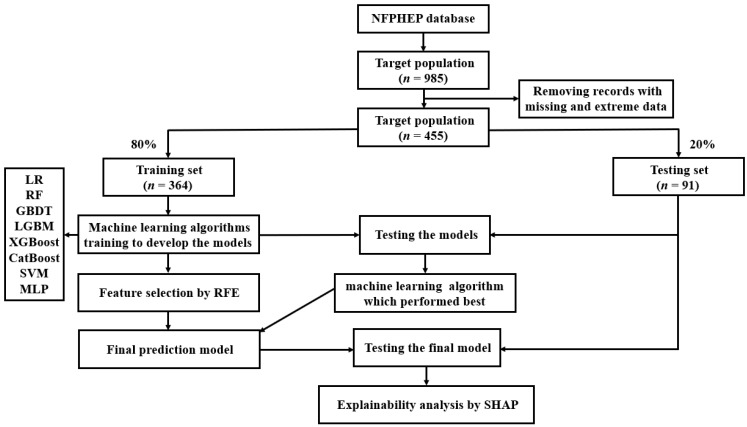
A flow chart of the methods used for data extraction, training, and testing. NFPHEP = National Free Preconception Health Examination Project, LR = logistic regression, RF = random forest, GBDT = gradient boosting decision tree, LGBM = light gradient boosting machine, XGBoost = extreme gradient boosting, CatBoost = category boosting, SVM = support vector machine, MLP = multi-layer perceptron, RFE = recursive feature elimination, SHAP = Shapley Additive Explanation.

The data processing flow is shown in Figure 1. All analyses were developed in Python (version 3.8.5). The dataset was divided randomly into the training set (80%, *n* = 364) and the testing sets (20%, *n* = 91) for the development and validation of the ML algorithms, respectively. Initially, 153 related features (Table S1) were included in ML as candidate variables for predictors. In the current study, eight ML algorithms were applied to develop the predictive models. The performances of the eight ML algorithms were evaluated by sensitivity, specificity, positive predictive value (PPV), negative predictive value (NPV) and AUC. Another measure of the quality of binary classification, Matthew’s correlation coefficient (MCC), was also evaluated, which is not affected by heavily imbalanced classes. Its value ranges from −1 to 1, where the random classification has a value of 0, the perfect classification has a value of 1, and the “completely wrong” classification has a value of −1. Furthermore, Cohen’s kappa was evaluated, which is another metric estimating the overall model performance. The AUC metric results were taken as the main index to measure the performances of the ML algorithms.

Being the best performing model, the extreme gradient boosting (XGBoost) algorithm was chosen for the final prediction model. In order to reduce the computational cost of modeling, 15 features which contributed greatly to the prediction were selected from 153 features by recursive feature elimination (RFE) to reduce the number of variables in the prediction model, incorporating a XGBoost classifier as the estimator. The effectiveness of RFE approach has been proven in various medical data [29,30,31]. A 5-fold cross-validation was performed to select the 15 most important features. These 15 features were included in the final prediction model using the ML algorithm which performed best among the eight algorithms. Grid search was employed for the hyperparameter tunning, and the employed hyperparameters of the best performed ML algorithm (XGBoost) were max depth = [(2, 3, 4, 5, 6, 7, 8), min child weight = (1, 2, 3, 4, 5, 6) and gamma = (0.5, 1, 1.5, 2, 5). The characteristics of the final model used in the hyperparameter tunning were booster = gbtree, gamma = 1, importance type = gain, learning rate = 0.01, max depth = 6, min child weight = 1, random state = 0, reg alpha = 0, reg lambda = 1.

Furthermore, in order to correctly interpret the ML prediction model, we applied post hoc explainability on the final model using the XGBoost algorithm, based on the Shapley Additive Explanation (SHAP) model, to explain the influence of all features included for model prediction. SHAP is a game theory approach which can evaluate the importance of individual input features to the prediction of a given model [32].

### 2.4. ML Algorithms

A conventional logistic regression (LR) method and seven popular ML classification algorithms, including random forest (RF), gradient boosting decision tree (GBDT), XGBoost, light gradient boosting machine (LGBM), category boosting (CatBoost), support vector machine (SVM) and multi-layer perceptron (MLP), were applied in the current study to model the data. All these algorithms are the most popular and up-to-date supervised ML methods for the problem of classification. The LR model is used to predict the probability of the binary dependent variable using a sigmoid function to determine the logistic transformation of the probability [33]. RF is an ensemble classification algorithm that combines multiple decision trees by majority voting [34,35]. GBDT is based on the ensembles of decision trees, which is popular for its accuracy, efficiency and interpretability. A new decision tree is trained at each step to fit the residual between ground truth and current prediction [36]. Many improvements have been made on the basis of GBDT. LGBM aggregates gradient information in the form of a histogram, which significantly improves the training efficiency [37]. CatBoost proposes a new strategy to deal with categorical features, which can solve the problems of gradient bias and prediction shift [38]. XGBoost is an optimized distributed gradient boosting library designed for speed and performance. It uses the second-order gradient, which is improved in the aspects of the approximate greedy search, parallel learning and hyperparameters [39]. SVM is a supervised learning model which targets to create a hyperplane. The hyperplane is a decision boundary between two classes, enabling the prediction of labels from one or more feature vectors. The main goal of SVM is to maximize the distance between the closest points of each class, called support vectors [40,41]. MLP is based on a supervised training process to generate a nonlinear predictive model, which belongs to the category of artificial neural network (ANN) and is the most common neural network. It consists of multiple layers such as input layer, output layer and hidden layer. Therefore, MLP is a hierarchical feed-forward neural network, where the information is unidirectionally passed from the input layer to the output layer through the hidden layer [42].

### 2.5. Statistical Analyses

Categorical variables were described as number (%) and compared by Chi-square or Fisher’s exact test where appropriate. Continuous variables that satisfy normal distribution were described as mean (standard deviation [SD]) and compared by the 2-tailed Student’s t-test; otherwise, median (interquartile range [IQR]) and Wilcoxon Mann–Whitney U test were used. The sensitivity, specificity, PPV, NPV, MCC and kappa of the models were calculated. The predictive power of the ML models was measured by AUC in the training and testing datasets. A two-sided *p* value < 0.05 was considered statistically significant. All statistical analyses were performed with Python (version 3.8.5).

## 3. Results

### 3.1. Baseline Characteristics

Of the 455 newborns whose mothers had been exposed to radiation in their living or working environment before pregnancy from 1 January 2010 to 31 December 2012 in the NFPHEP database, a total of 60 SGA births occurred (13.2%). Demographic characteristics of the study population are shown in Table 1. Supplementary Table S1 lists the results comparing the 153 candidate variables for predictors in the study cohort. Overall, the median gestational age of the newborns in the cohort was 40.0 weeks (IQR, 39.0–40.0). The birth weight of SGA newborns (2.6 kg [2.2–2.8]) was significantly lower than that of non-SGA newborns (3.4 kg [3.1–3.6]). Maternal height was significantly lower in the SGA newborns compared to the non-SGA newborns (158.0 cm [155.0–160.0] versus 160.0 cm [157.0–163.0]). The mothers of SGA newborns had a significantly higher incidence of adnexitis before pregnancy (15.0% vs. 3.5%) compared to the mothers of non-SGA newborns. In addition, the number of previous pregnancies in the mothers of SGA newborns was significantly higher than those of non-SGA newborns. Furthermore, the fathers of SGA newborns had a significantly higher incidence of anemia (8.3% vs. 1.3%) compared with those of non-SGA newborns.

### 3.2. ML Algorithms’ Performance Comparison

**Figure 2:**
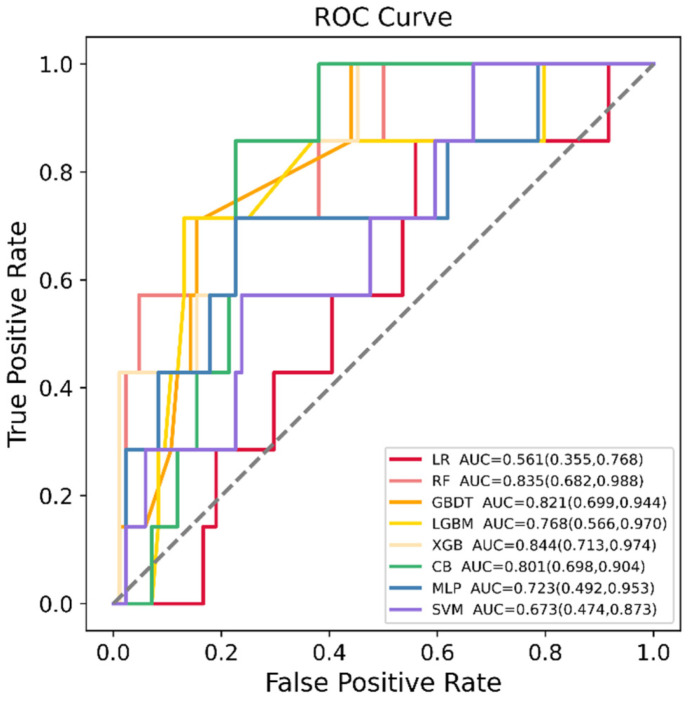
Receiver operating characteristic (ROC) curves of the eight machine learning (ML) models in predicting small for gestational age (SGA) in the testing dataset. LR = logistic regression, RF = random forest, GBDT = gradient boosting decision tree, LGBM = light gradient boosting machine, XGB = extreme gradient boosting, CB = category boosting, MLP = multi-layer perceptron, SVM = support vector machine.

LR, RF, GBDT, XGBoost, LGBM, CatBoost, SVM and MLP were developed in the training dataset (*n* = 364), and their SGA prediction performance was compared in the testing dataset (*n* = 91). Figure 2 shows the ROC curve comparison of the developed models in the testing dataset for SGA prediction. Overall, the model obtained by XGBoost achieved the highest AUC value in the testing set, 0.844 [95% confidence interval (CI): 0.713–0.974]. All models showed a good AUC for predicting SGA: XGBoost (AUC: 0.844, 95% CI: 0.713–0.974), RF (AUC: 0.835, 95% CI: 0.682–0.988), GBDT (AUC: 0.821, 95% CI: 0.699–0.944), CatBoost (AUC: 0.801, 95% CI: 0.698–0.904), LGBM (AUC: 0.768, 95% CI: 0.566–0.970), MLP (AUC: 0.723, 95% CI: 0.492–0.953) and SVM (AUC: 0.673, 95% CI: 0.474–0.873), except for LR (AUC: 0.561, 95% CI: 0.355–0.768). In addition, the AUC values in the training set and testing set, sensitivity, specificity, PPV, NPV, MCC and kappa values of each model are listed in Table 2. Model sensitivity, specificity, PPV, NPV, MCC and kappa ranged from 0.714 to 1.000, 0.333 to 0.869, 0.111 to 0.312, 0.970 to 1.000, 0.161 to 0.408 and 0.071 to 0.367, respectively.

### 3.3. Feature Selection and Final Prediction Model

**Figure 3:**
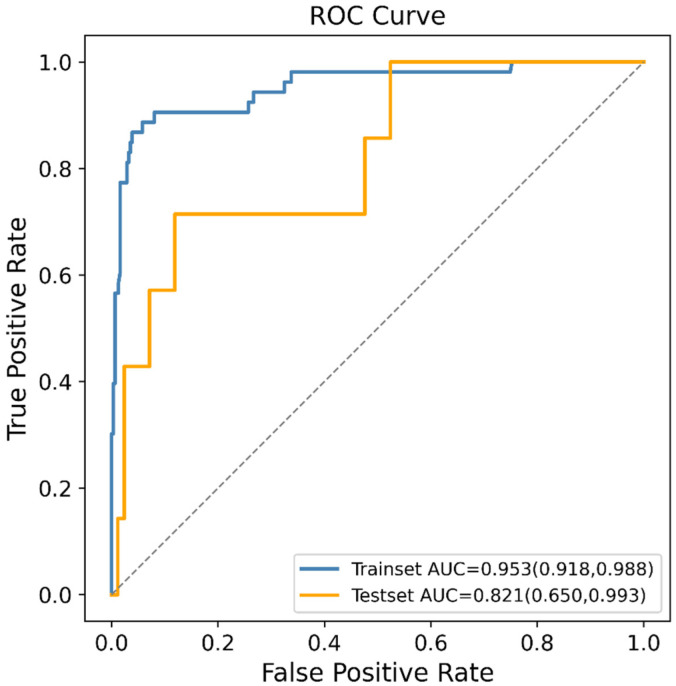
Receiver operating characteristic (ROC) curves of the final machine learning (ML) model generated after recursive feature elimination (RFE) in predicting small for gestational age (SGA).

In order to reduce the computational cost of modeling, 15 features which contributed greatly to the prediction were selected from 153 features by the RFE method. These features were maternal adnexitis before pregnancy, maternal body mass index (BMI) before pregnancy, maternal systolic blood pressure before pregnancy, maternal education level, maternal platelet count (PLT) before pregnancy, maternal blood glucose before pregnancy, maternal alanine aminotransferase (ALT) before pregnancy, maternal creatinine before pregnancy, paternal drinking before pregnancy, paternal economic pressure before pregnancy, paternal systolic blood pressure before pregnancy, paternal diastolic blood pressure before pregnancy, paternal ALT before pregnancy, maternal PM2.5 exposure in the first trimester and maternal PM2.5 exposure in the last trimester. These 15 features were included in the final prediction model using the XGBoost algorithm which exhibited the highest AUC value in the previous model comparison. Figure 3 shows the ROC curve of the final prediction model in the training and testing dataset for SGA prediction. The AUC values in the training set and testing set, sensitivity, specificity, PPV, NPV, MCC and kappa values of the final model were 0.953 (95% CI: 0.918–0.988), 0.821 (95% CI: 0.650–0.993), 0.714, 0.881, 0.333, 0.974, 0.427 and 0.391, respectively, proving the superiority of the feature selection approach and the employed ML algorithm.

### 3.4. Assessment of Variable Importance

**Figure 4:**
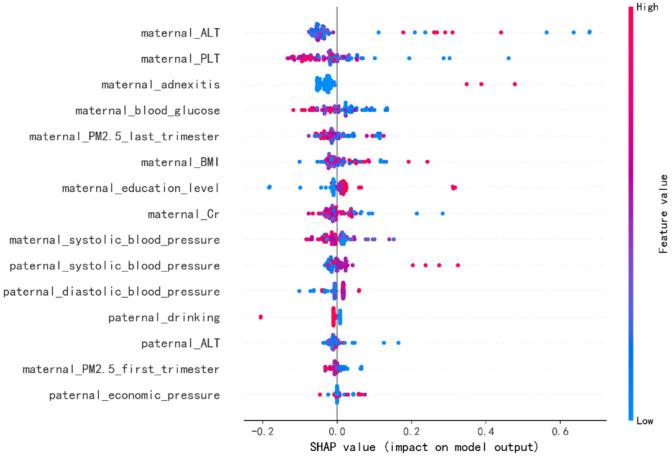
The Shapley Additive Explanation (SHAP) values for most important predictors of small for gestational age (SGA) in the final model. ALT = alanine aminotransferase, PLT = platelet count, BMI = body mass index, Cr = creatinine. Each line represents a feature, and the abscissa is the SHAP value, which represents the degree of influence on the outcome. Each dot represents a sample. Plot is colored red (blue) if the value of the feature is high (low).

**Figure 5:**
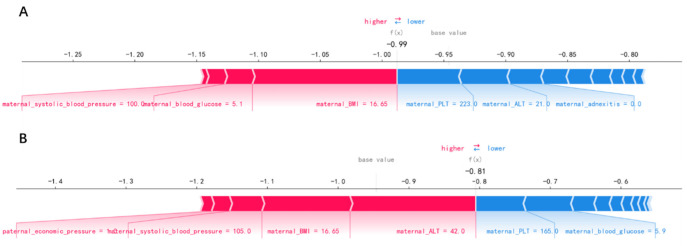
Newborns correctly classified as non-small-for-gestational-age (**A**) and small-for-gestational-age (**B**).

In order to identify the features that had the greatest impact on the final prediction model (XGBoost), we drew the SHAP summary diagram of the final prediction model (Figure 4). The feature names were plotted on the *y*-axis from top to bottom according to their importance, while the *x*-axis represented the mean SHAP values. Each dot represented a sample. Plot was colored red (blue) if the value of the feature was high (low). The 6 most important features for the SGA prediction were maternal ALT before pregnancy, maternal PLT before pregnancy, maternal adnexitis before pregnancy, maternal blood glucose before pregnancy, maternal PM2.5 exposure in the last trimester and maternal BMI before pregnancy. In addition, Figure 5 shows two examples for newborns that were classified correctly as non-SGA and SGA, respectively.

## 4. Discussion

This study represents the first report using ML algorithms in the development and validation of a risk prediction model for SGA newborns in pregnant women exposed to radiation before pregnancy. Additionally, paternal risk factors and maternal PM2.5 exposure during pregnancy were innovatively included in our ML models as predictive features. Our study demonstrates that ML algorithms can yield more effective prediction models than the conventional logistic regression, and the XGBoost model exhibited the best performance for SGA prediction (AUC: 0.844), suggesting that ML is a promising approach in predicting SGA newborns. With our models, the antenatal prediction of SGA could be made to monitor at-risk fetuses more closely and improve perinatal outcomes.

Evidence indicated that the SGA proportions increased with the radiation exposure [8,9]. Females who have received abdominal or pelvic radiation, radiation for their childhood cancer and diagnostic radiography for idiopathic scoliosis experienced an increased risk of low birth weight among their offspring [12,43,44,45]. Low birth weight has been considered to be an indicator of genetic damage caused by mutations in humans exposed to radiation [46]. However, to our knowledge, no study has established a prediction model for SGA newborns in women exposed to radiation before pregnancy. In our study, eight ML models were used for a comparative evaluation (Table 2). Among these models, XGBoost, RF, GBDT and CatBoost showed similar performance based on the AUC value, with XGBoost having the highest AUC value (0.844). However, the LR model had the lowest AUC value of 0.561. This might be due to the fact that the LR algorithm is sensitive to outliers and requires a large dataset to work well. Additionally, the imbalanced dataset may affect the performance of the LR model. The results of our study indicated that the ML algorithm was a promising approach to predict SGA newborns in women exposed to radiation before pregnancy, with superior discrimination than the conventional LR (AUC: 0.844 versus 0.561).

Only based on 15 features including the demographic characteristics of parents, simple and feasible clinical test indexes and regional PM2.5 exposure, an effective SGA prediction model could be established (AUC: 0.821, Figure 3), indicating that the appropriate features were selected from 153 features by RFE approach. The RFE algorithm is a wrapper-based backward elimination process by recursively computing the learning function, performing a recursive ranking of a given feature set [47]. Its effectiveness has been extensively proven in various medical data [29,30,31,48]. Recently, a new ensemble feature selection methodology has been proposed, which aggregates the outcomes of several feature selection algorithms (filter, wrapper and embedded ones) to avoid bias [49,50]. The robust feature selection methodology can be applied in future work. Additionally, advanced ML algorithms provided great potential for improving SGA prediction. The reason was that the interactions between predictors might exist but were not detected by conventional modeling methods. Such weakness could be remedied with the advanced ML algorithms explored in our current study. The ability of ML algorithms to automatically process multidimensional and multivariate data could eventually reveal novel associations between specific features and the SGA outcome and identify trends that would be unobvious to researchers otherwise [51].

Paternal risk factors and maternal PM2.5 exposure during pregnancy were included in the ML prediction models for SGA newborns for the first time. Mounting studies have been devoted to identifying maternal risk factors for the adverse birth outcomes. Little attention has been paid to the fact that paternal factors could also predict adverse birth outcomes. Several paternal factors have been confirmed as risk factors for SGA newborns, such as paternal age, height, ethnicity, education level and smoking during pregnancy [21,22,52,53,54]. Moreover, women exposed to excessive PM2.5 during pregnancy also had an increased risk of delivering SGA offspring [23]. However, these factors have not been considered in the previous SGA prediction models established in the general population. The results of our study demonstrated that paternal drinking, economic pressure, blood pressure and ALT, maternal PM2.5 exposure in the first trimester and last trimester were all included in the top 15 most contributing features, suggesting that the paternal factor and maternal PM2.5 exposure during pregnancy were involved in the risk prediction for SGA in the study population.

Figure 4 showed the features’ impact on the output of the final model (XGBoost). The SHAP values were used to represent the impact distribution of each feature on the model output. For instance, a low maternal PLT level increased the predicted status of the subjects. The features maternal blood glucose, creatinine and systolic blood pressure presented a similar behavior. In contrast to that, maternal adnexitis, high education level and high paternal blood pressure had a positive effect on the prediction outcome. The top 6 most influential features in the SHAP summary plot of the final prediction model were maternal ALT, PLT, adnexitis, blood glucose, PM2.5 exposure in the last trimester and BMI before pregnancy. In addition to the known risk factor maternal PM2.5 exposure, recent studies showed that reduced fetal growth was associated with increased maternal ALT [55]. The significant association between maternal PLT and adverse perinatal outcome has been reported [56]. Additionally, pelvic inflammatory diseases have been linked to adverse perinatal outcomes including SGA [57,58]. In addition, maternal blood glucose and pre-pregnancy BMI have been reported to be associated with increased risk of delivering SGA infants [59,60,61], which is consistent with our findings. Changes in these features caused by radiation exposure also have been reported in previous studies [62,63,64,65]. In addition, using SHAP force plots, two examples that were classified correctly as non-SGA and SGA were selected to explain the effects of the features on the prediction outcome (Figure 5). The contribution of each feature to the output result was represented by an arrow, the force of which was related to the Shapley value. They showed how each feature contributed to push the model output from the baseline prediction to the corresponding model output. The red arrows represented features increasing the predicted results. The blue arrows represented features decreasing the predicted results. It was observed that lower values of maternal BMI, blood glucose, systolic blood pressure and higher values of maternal ALT pushed the output prediction to the SGA class.

This study has several limitations. Firstly, although the data were collected nationally, the sample size was small which may indicate bias. With a larger sample size in the future work, a stratified k-fold cross validation can be used to improve the accuracy of the results. Secondly, there was a lack of the type and average daily exposure of the radiation in mothers’ living or working environment before pregnancy in the dataset. Moreover, ultrasound biometrics measurements were lacking in the dataset, and their inclusion in the prediction model may further improve the accuracy and applicability of the model. Further validation and application of ML into the daily clinical practice is still necessary to better understand its real value in predicting SGA newborns.

## 5. Conclusions

In this work, a comprehensive analysis of SGA newborns prediction in pregnant women exposed to radiation in their living or working environment before pregnancy was carried out, with the help of feature selection and optimization techniques. It is concluded that ML algorithms show good performances on the classification of SGA newborns. The final model using the XGBoost algorithm achieves effective SGA prediction (AUC: 0.821) only based on 15 features, including the demographic characteristics of parents, simple and feasible clinical test indexes and regional PM2.5 exposure. Furthermore, the post hoc analysis complemented the prediction results by enhancing the understanding of the contribution of the selected features to the classification of SGA newborns. ML models may be a potential assistant approach for the early prediction of delivering SGA newborns in high-risk populations. Future work aims to work with other ensemble feature selection methodologies and apply the proposed methodology to other high-risk populations for delivering SGA newborns.

## Figures and Tables

**Table 1:** Demographic characteristics of the subjects included in analysis.

Parameters	Overall (*n* = 455)	Not SGA (*n* = 395)	SGA (*n* = 60)	*p* Value
Gestational at birth, week	40.0 (39.0–40.0)	40.0 (39.0–40.0)	40.0 (39.0–40.0)	0.013
Birth weight, kg	3.3 (3.0–3.6)	3.4 (3.1–3.6)	2.6 (2.2–2.8)	<0.001
Maternal age, year	24.0 (23.0–27.0)	24.0 (23.0–27.0)	24.5 (22.0–26.0)	0.184
Maternal height, cm	160.0 (156.0–163.0)	160.0 (157.0–163.0)	158.0 (155.0–160.0)	0.014
Maternal BMI, kg/m^2^	20.2 (18.8–22.0)	20.2 (18.8–22.0)	20.0 (18.6–22.2)	0.332
Maternal education level				
Below junior high school	168 (36.9%)	149 (37.7%)	19 (31.7%)	0.635
Senior high school	146 (32.1%)	126 (31.9%)	20 (33.3%)	
Bachelor’s degrees and above	141 (31.0%)	120 (30.4%)	21 (35.0%)	
Mother adnexitis before pregnancy	23 (5.1%)	14 (3.5%)	9 (15.0%)	0.001
Number of previous pregnancies	0.0 (0.0–1.0)	0.0 (0.0–1.0)	1.0 (0.0–1.0)	0.003
Paternal age, year	26.0 (24.0–29.0)	26.0 (24.0–28.0)	26.0 (24.0–29.0)	0.328
Paternal height, cm	171.4 ± 5.3	171.6 ± 5.2	170.2 ± 5.6	0.055
Paternal education level				
Below junior high school	174 (38.2%)	153 (38.7%)	21 (35.0%)	0.810
Senior high school	151 (33.2%)	131 (33.2%)	20 (33.3%)	
Bachelor’s degrees and above	130 (28.6%)	111 (28.1%)	19 (31.7%)	
Father anemia before pregnancy	10 (2.2%)	5 (1.3%)	5 (8.3%)	0.003

SGA = small for gestational age, BMI = body mass index. Data are presented as median (interquartile range), mean (standard deviation) or number (%). Categorical variables are compared by Chi-square or Fisher’s exact test where appropriate. Continuous variables that satisfy normal distribution are compared by the 2-tailed Student’s *t*-test; otherwise, Wilcoxon Mann–Whitney U test are used.

**Table 2:** Performance of models by different algorithms in predicting small for gestational age (SGA) neonates.

Model	AUC Training	AUC Testing	Sensitivity	Specificity	PPV	NPV	MCC	Kappa
LR	0.620	0.561	0.857	0.440	0.113	0.974	0.161	0.074
RF	0.897	0.835	0.714	0.845	0.278	0.973	0.374	0.325
GBDT	0.850	0.821	0.714	0.845	0.278	0.973	0.374	0.325
XGBoost	0.958	0.844	0.857	0.774	0.240	0.985	0.377	0.290
LGBM	0.844	0.768	0.714	0.869	0.312	0.973	0.408	0.367
CatBoost	0.853	0.801	0.857	0.774	0.240	0.985	0.377	0.290
SVM	0.836	0.673	1.000	0.333	0.111	1.000	0.192	0.071
MLP	0.902	0.723	0.714	0.774	0.208	0.970	0.295	0.231

AUC = area under the receiver-operating-characteristic curve, PPV = positive predictive value, NPV = negative predictive value, MCC = Matthews correlation coefficient, LR = logistic regression, RF = random forest, GBDT = gradient boosting decision tree, XGBoost = extreme gradient boosting, LGBM = light gradient boosting machine, CatBoost = category boosting, SVM = support vector machine, MLP = multi-layer perceptron.

## Data Availability

Our research data were derived from the National Free Preconception Health Examination Project (NFPHEP). Requests to access these datasets should be directed to Hui Pan, panhui20111111@163.com.

## Supplementary Materials

The following supporting information can be downloaded at: https://www.mdpi.com/article/10.3390/jpm12040550/s1, Table S1: 153 features included in machine learning models as candidate variables for predictors.
